# Sleep fragmentation induces heart failure in a hypertrophic cardiomyopathy mouse model by altering redox metabolism

**DOI:** 10.1016/j.isci.2024.109075

**Published:** 2024-02-01

**Authors:** Karthikeyan Bose, Radhika Agrawal, Thiagarajan Sairam, Jessenya Mil, Matthew P. Butler, Perundurai S. Dhandapany

**Affiliations:** 1 The Knight Cardiovascular Institute and Departments of Medicine, Molecular, and Medical Genetics, Oregon Health and Science University, Portland, OR 97239, USA; 2 Cardiovascular Development and Disease Mechanisms, Institute for Stem Cell Science and Regenerative Medicine, Bangalore (DBT-inStem), Bangalore, India; 3 Oregon Institute of Occupational Health Sciences, and Department of Behavioral Neuroscience, Oregon Health and Science University, Portland, OR 97239, USA

**Keywords:** Cardiovascular medicine, Physiology, Molecular biology

## Abstract

Sleep fragmentation (SF) disrupts normal biological rhythms and has major impacts on cardiovascular health; however, it has never been shown to be a risk factor involved in the transition from cardiac hypertrophy to heart failure (HF). We now demonstrate devastating effects of SF on hypertrophic cardiomyopathy (HCM). We generated a transgenic mouse model harboring a patient-specific myosin binding protein C3 (MYBPC3) variant displaying HCM, and measured the progression of pathophysiology in the presence and absence of SF. SF induces mitochondrial damage, sarcomere disarray, and apoptosis in HCM mice; these changes result in a transition of hypertrophy to an HF phenotype by chiefly targeting redox metabolic pathways. Our findings for the first time show that SF is a risk factor for HF transition and have important implications in clinical settings where HCM patients with sleep disorders have worse prognosis, and strategic intervention with regularized sleep patterns might help such patients.

## Highlights

• Sleep fragmentation (SF) induces heart failure (HF) in hypertrophic cardiomyopathy (HCM)
• SF causes structural and biochemical changes in HCM
• SF affects redox metabolism in HCM leading to HF
• Regularized sleep patterns might help HCM patients

## Graphical abstract

**Figure:**
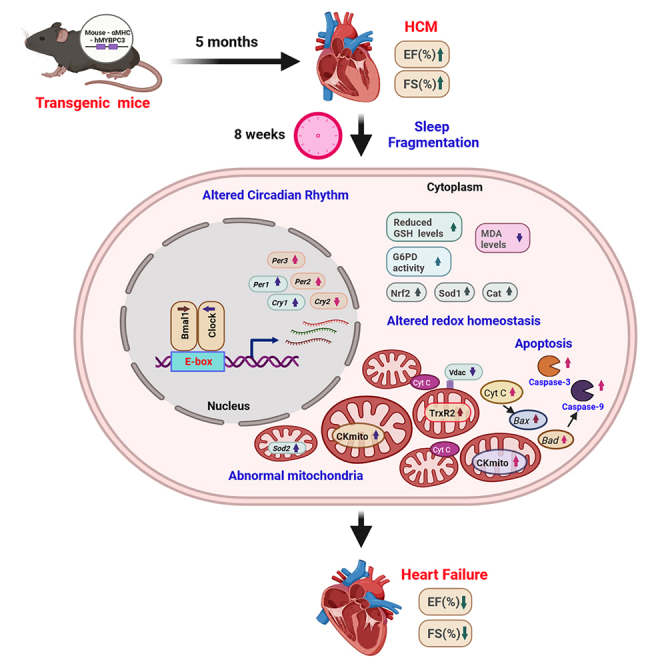

## Introduction

Sleep is a fundamental physiological process^1^ and plays a crucial role in cardiovascular health.^2^^,^^3^^,^^4^ Sleep fragmentation (SF) or deprivation exacerbates various disease conditions including obesity,^5^ diabetes,^6^ cancer,^7^ brain^8^ and cardiovascular diseases.^9^ Specifically, SF has a strong negative effect on cardiovascular health by increasing sympathetic tone.^10^ Recent human studies have demonstrated that night shift work augments the risk for cardiovascular disease potentially via disruption of the sleep/wake cycle.^11^ In fact, obstructive sleep apnea (OSA) and central sleep apnea (CSA), two common sources of SF, are frequently observed in heart failure (HF) patients and increase the morbidity and mortality in these patients.^10^

Hypertrophic cardiomyopathy (HCM) due to abnormal ventricular remodeling is predominantly caused by sarcomere gene variants (e.g., *MYBPC3* and *MYH7* variants).^12^ Over time, HCM can transit and worsen to dilation and end-stage HF assisted by various factors.^13^ SF is one of the widely suspected contributing factors in the transition of HCM to HF.^10^^,^^14^ However, to date, no studies have shown the influence of SF on a genetically predisposed HCM model and its related mechanisms in disease progression.

To address these issues, we generated and characterized a new transgenic mouse model carrying a modified C10 domain of the human MYBPC3 protein that mimics the effects of a 25 bp deletion causing HCM.^15^^,^^16^^,^^17^ In particular, we have shown that the MYBPC3 25 bp deletion is common in human populations and is associated with cardiomyopathy that affects approximately 100 million people worldwide.^15^^,^^16^ In the HCM transgenic mice, we tested the hypothesis that SF would have adverse effects on heart health. After eight weeks of SF, a rapid transition toward dilated/HF phenotypes was observed in HCM mouse hearts. We show that the rapid HF transition is due to mitochondrial dysfunction, apoptosis, and subsequent dysregulation of redox balance in SF-subjected HCM mouse hearts.

## Results

### Transgenic humanized MYBPC3 mouse model exhibits HCM phenotypes

**Figure 1:**
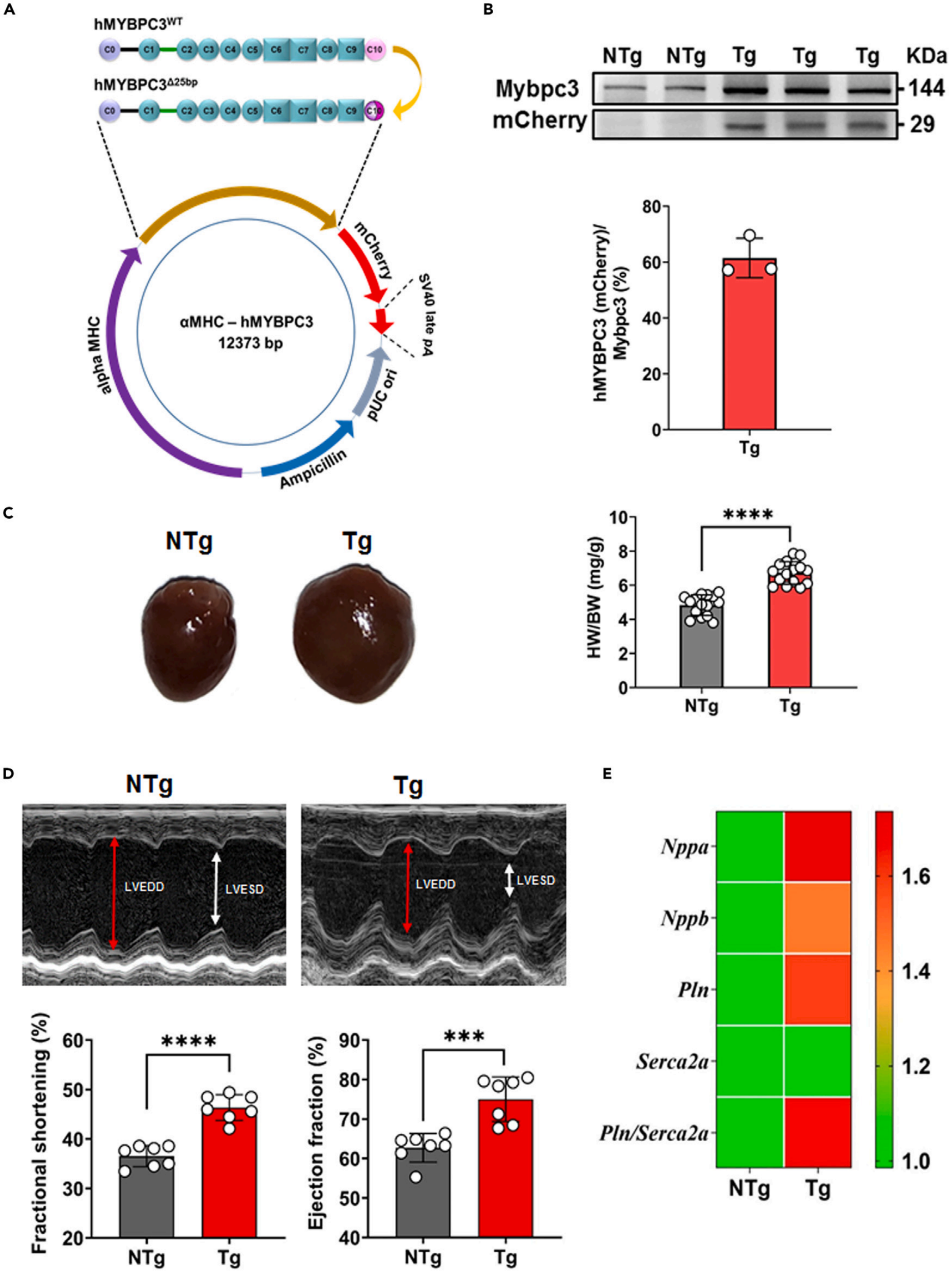
Generation and characterization of genetically predisposed HCM mice (A) Cloning strategy for generating genetically predisposed HCM mouse model harboring human patient-specific *MYBPC3* 25bp deletion (hMYBPC3^Δ25bp^) under the control of mouse αMHC promoter. (B) Representative immunoblot showing levels of Mybpc3 and hMYBPC3^Δ25bp^ (mCherry) transgene. Protein expression analysis of hMYBPC3^Δ25bp^ (mCherry) transgene in comparison with total Mybpc3 in Tg mice heart. Expression levels were normalized to internal loading control (Gapdh) and presented as percentage levels. (C) Cardiac phenotype of transgenic (Tg) and non-transgenic (NTg) mice at an age of five months and heart weight/body weight (HW/BW) ratio showing hypertrophy in Tg compared to NTg mice. (D) Representative M-mode echocardiograms from NTg and Tg mice and percentage of ejection fraction and fraction shortening in NTg and Tg mice at 5 months of age, respectively (n = 7 in each group). Red and white arrow indicates the left ventricular end-diastolic diameter (LVEDD) and left ventricular end-systolic diameter (LVESD), respectively. (E) Quantitative RT-PCR analysis of hypertrophic marker (*Nppa* and *Nppb*) and calcium handling (*Pln* and *Serca2a*) genes and their ratios in Tg and NTg mice heart. mRNA levels were normalized to *Gapdh* and presented as relative expression levels compared to levels in NTg mice heart. Values are mean ± SEM with each experiment performed in triplicates (n = 6 in each group). Significance was evaluated by Student’s *t* test. ∗∗∗p < 0.001 and ∗∗∗∗p < 0.0001.

Previously, we showed that a 25 bp deletion in the *MYBPC3* (*MYBPC3*^*Δ25bp*^) gene affecting the C10 domain of the MYBPC3 protein is associated with cardiomyopathy.^16^ To obtain an HCM model, a cardiac-specific transgenic mouse model overexpressing ∼50–60% of *MYBPC3* mutant proteins with a modified C10 domain was generated (Tg-low; hereafter designated Tg as outlined in the methods, Figures 1A and 1B). Our immunoblotting analysis showed that the mutated protein expression (Figure 1B) was similar to that in other existing *MYBPC3* mutant mouse HCM models, suggesting a poison polypeptide mechanism.^17^ The Tg mice developed cardiac hypertrophy at 5 months and recapitulated HCM patient phenotypes, including an increase in heart weight to body weight ratio (Figure 1C). To evaluate cardiac function in Tg mouse hearts compared with non-transgenic mice (NTg) controls, echocardiography was performed using ultrasound imaging. As shown in Figures 1D and 1E, the Tg mouse hearts displayed significant elevations in the ejection fraction (EF), fractional shortening (FS), and expression of fetal genes (*Nppa* and *Nppb*) and calcium handling gene ratios (*Pln/Serca2a*) compared to NTg controls. These data suggest that the transgenic mouse model displayed features of HCM.

### Sleep fragmentation induces heart failure phenotypes

**Figure 2:**
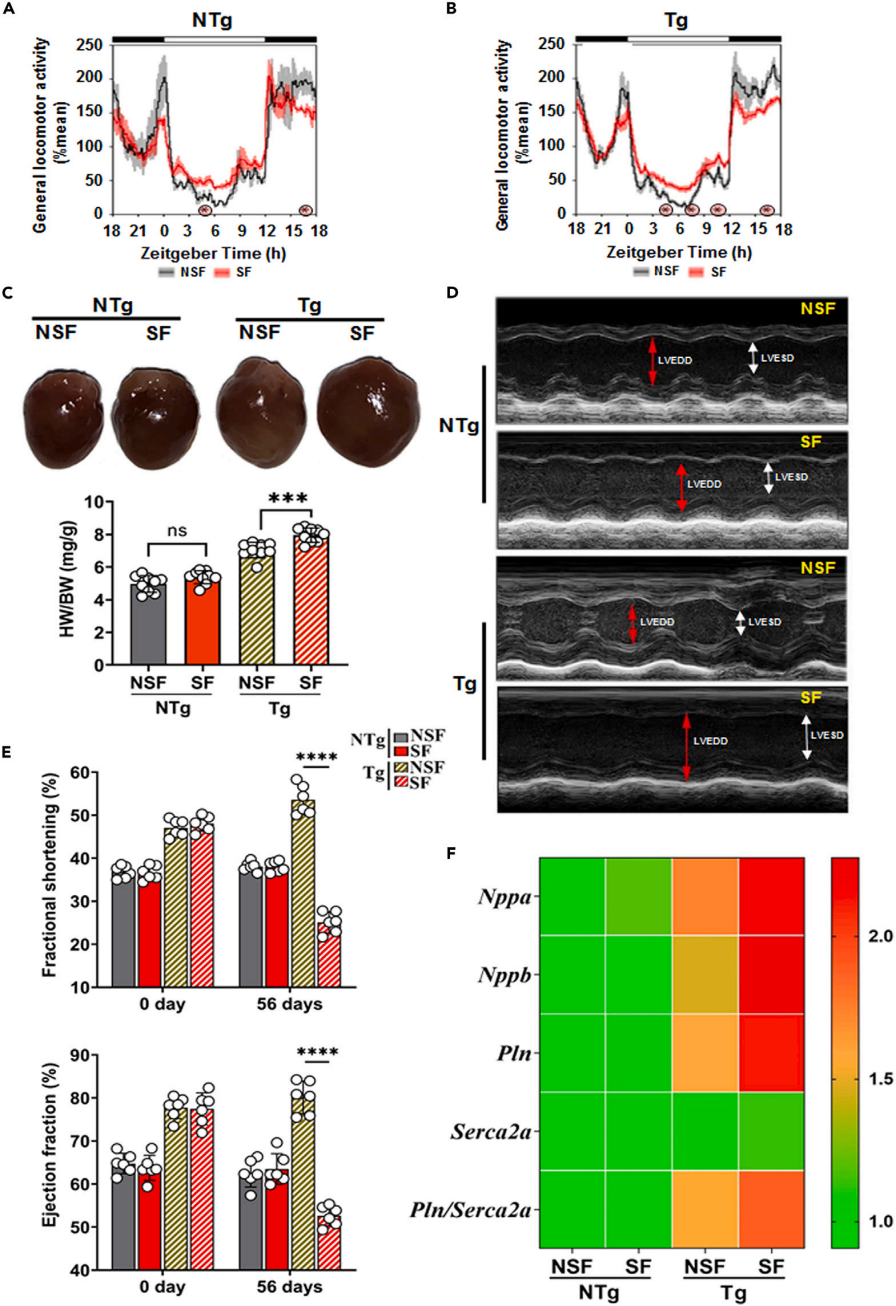
SF induce heart failure (HF) phenotypes (A and B) Spontaneous locomotor activity collected in 10 min bins was normalized as percent of the daily mean within the cage, and then profiles (mean and SE) were plotted. White and black bar at the top indicates the light-dark cycle, with lights-on at zeitgeber time 0 (ZT0) and lights-off at ZT12. Significantly elevated activity during the normal resting phase suggests fragmentation of sleep. ∗p <0 .05, Tukey test between condition for collapsed 3 h bins. (C) Gross heart morphology and heart weight/body weight (HW/BW) ratio of NTg-NSF, NTg-SF, Tg-NSF, and Tg-SF mice. (D and E) Representative M-mode echocardiography and percentage of EF and FS in NTg-NSF, NTg-SF, Tg-NSF, and Tg-SF mice (n = 6 in each group). Red and white arrow indicates the left ventricular end-diastolic diameter (LVEDD) and left ventricular end-systolic diameter (LVESD), respectively. (F) Quantitative RT-PCR analysis of hypertrophic markers (*Nppa* and *Nppb*) and calcium handling (*Pln* and *Serca2a*) genes and their ratios in NTg-NSF, NTg-SF, Tg-NSF, and Tg-SF mouse hearts. mRNA levels were normalized to *Gapdh* and presented as relative expression levels compared to levels in NTg-NSF mice heart. Values are mean ± SEM with each experiment performed in triplicates (n = 6 in each group). Significance was evaluated by Student’s *t* test or one-way analysis of variance (ANOVA) with post hoc sidak multiple comparison test, respectively. ∗∗∗p < 0.001 and ∗∗∗∗p < 0.0001. See also Figure S1.

To assess whether SF influences the HCM disease course, NTg without HCM and littermate-matched Tg mice with HCM were subjected to SF for eight weeks and compared with their respective non-sleep fragmented mice (NTg-NSF and Tg-NSF mice) (Figures 2A and 2B; Figure S1). The Tg-SF mice displayed an increased heart to body weight ratio compared with Tg-NSF mice. In contrast, SF did not affect heart size in NTg mice (Figure 2C).

Next, we assessed the cardiac function of all the experimental mice through echocardiographic M-mode analyses. There were no significant effects on the systolic function of the heart observed in the NTg-SF mice compared to NTg-NSF mice. In contrast, Tg-SF mouse hearts showed altered systolic functions with an increased left ventricular end-systolic diameter (LVESD) and left ventricular end-diastolic diameter (LVEDD) (Figure 2D) and a decreased FS and EF (Figure 2E) compared to Tg-NSF mice. The expression levels of pathological gene markers, including *Nppa*, *Nppb*, and calcium handling gene ratios (*Pln/Serca2a*), were higher in Tg-SF mouse hearts than in Tg-NSF mouse hearts (Figure 2F). Together, these results suggest that SF can induce left ventricular (LV) chamber dilatation and elevate pathological gene markers in Tg mice, which indicates a transition from initial hypertrophy to dilated/HF phenotypes.

### Expression of circadian genes is altered due to SF

**Figure 3:**
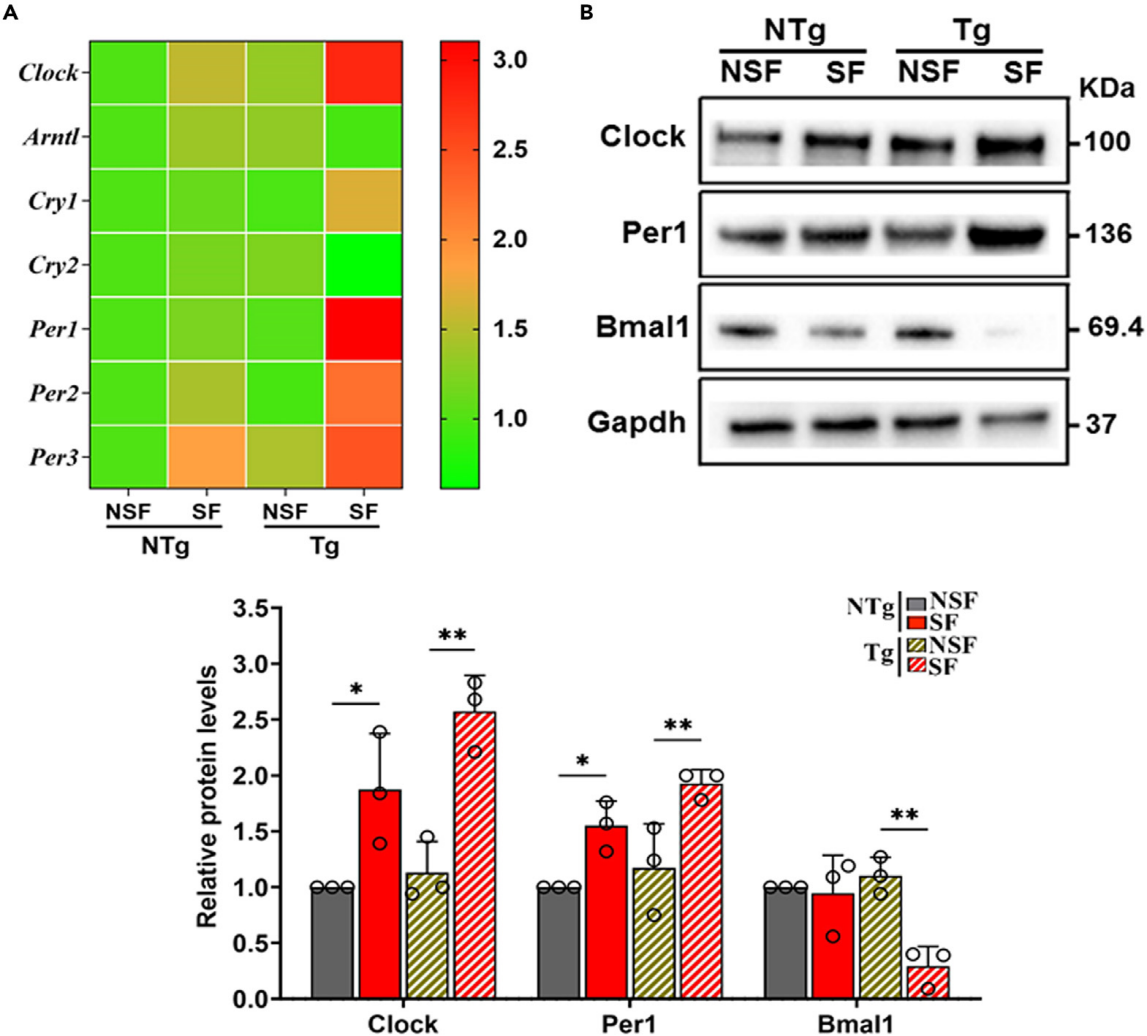
SF alters the expression of circadian genes (A) Quantitative RT-PCR analysis of core circadian genes (*Clock*, *Arnt1*(*Bmal1*), *Cry1*, *Cry2*, *Per1*, *Per2*, and *Per3*) in NTg-NSF, NTg-SF, Tg-NSF, and Tg-SF mouse heart tissues. mRNA levels were normalized to *Gapdh* and presented as relative expression levels compared to level in NTg-NSF mouse hearts. Values are mean ± SEM with each experiment performed in triplicates (n = 6 in each group). (B) Representative immunoblots with respective proteins from the total lysates of mouse heart tissues isolated from NTg-NSF, NTg-SF, Tg-NSF, and Tg-SF mouse heart tissues. Expression levels were normalized to loading control and presented as relative expression levels compared with the level in NTg-NSF mouse heart. Gapdh levels were used as a loading control. Values are shown as means ± SEM with each experiment performed in triplicate (n = 3 in each group). Significance was evaluated by one-way analysis of variance (ANOVA) with post hoc sidak multiple comparison test, respectively. ∗p < 0.05 and ∗∗p < 0.01.

Sleep disturbance is known to alter the expression of circadian core clock genes in various diseases.^18^^,^^19^ To understand the influence of chronic SF on circadian genes, we analyzed the expression profile of critical clock genes (*Clock*, *Arntl* (*Bmal1*), *Cry1*, *Cry2*, *Per1*, *Per2* and *Per3*) in all the experimental groups of mice. When compared to that of NSF-NTg mice, the expression levels of most clock genes studied were slightly upregulated in SF-exposed NTg mice (Figure 3A). In contrast, the differential expression levels of the clock genes were more pronounced in the Tg-SF mouse hearts. These mouse hearts in this group exhibited increases in the expression levels of *Clock*, *Cry1*, *Per1*, *Per2*, and *Per3* and decreases in the expression levels of the *Arntl* (*Bmal1*) and *Cry2* genes compared to those in the Tg-NSF group or any other experimental groups (Figure 3A).

Our immunoblot analysis showed increases in Clock and Per1 and decreases in Bmal1 at the protein level in both NTg-SF and Tg-SF mouse hearts compared to the respective NTg-NSF and Tg-NSF mouse hearts (Figure 3B). Notably, Tg-SF mice showed a more pronounced increase in Clock and Per1 levels and decrease in Bmal1 levels than NTg-SF mice (Figure 3B). These results suggest that SF affects normal cardiac circadian gene expressions in Tg-SF mouse hearts.

### Mitochondrial dysfunction and apoptosis in mouse hearts with SF

**Figure 4:**
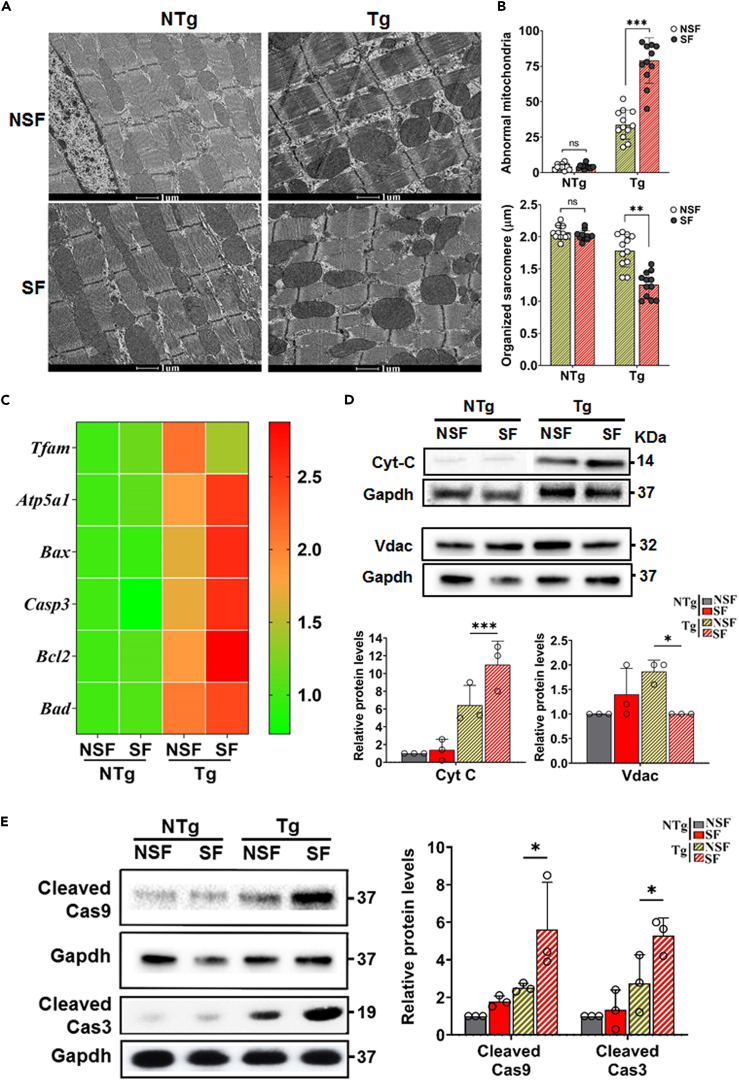
SF alters mitochondrial function and apoptosis related gene expression (A and B) Representative TEM micrographs and quantification of abnormal hypertrophied and rounded mitochondria and sarcomere length in NTg-NSF, NTg-SF, Tg-NSF, and Tg-SF mouse heart tissues, respectively. Data shown as mean ± SEM, n = 187 sarcomeres from three mice in each group. (C) Quantitative RT-PCR analysis of mitochondrial stability (*Tfam* and *Atp5a*) and apoptosis (*Bax*, *Cas3*, *Bcl2*, and *Bad*) related genes in mouse heart tissues. mRNA levels were normalized to *Gapdh* and presented as relative expression levels compared to levels in NTg-NSF mice heart. Values are mean ± SEM with each experiment performed in triplicates (n = 6). (D and E) Representative immunoblots with respective proteins from the total lysates of mouse heart tissues isolated from NSF and SF exposed NTg and Tg mice, respectively. Expression levels were normalized to loading control and presented as relative expression levels compared with the level in NTg-NSF mouse hearts. *Gapdh* levels were used as a loading control. Values are shown as means ± SEM with each experiment performed in triplicate (n = 3 in each group). Significance was evaluated by Mann-Whitney U test or one-way analysis of variance (ANOVA) with post hoc sidak multiple comparison test, respectively.∗p < 0.05, ∗∗p < 0.01, and ∗∗∗p < 0.001. See also Figure S2.

Sleep deprivation alters mitochondrial morphology, gene expression and oxidative phosphorylation activity in mice and humans.^20^^,^^21^^,^^22^^,^^23^^,^^24^^,^^25^^,^^26^ Hence, we studied mitochondrial structure by transmission electron microscopy (TEM) imaging and quantified the mRNA expression and protein levels of genes associated with mitochondrial functions in all experimental mouse hearts. TEM ultrastructure analysis of both NTg-NSF and SF mouse heart tissue sections showed no significant alterations in the mitochondrial structure of sarcomere alignments (Figures 4A and 4B). However, Tg-SF mouse heart tissue sections displayed an increased abnormal mitochondrial shape and disorganization of the sarcomere pattern relative to those of Tg-NSF mice. In addition, Tg-SF mouse heart sections displayed a significant decrease in sarcomere alignment compared with Tg-NSF mouse heart sections (Figures 4A and 4B). Notably, in Tg-SF mouse hearts, we observed a decrease in the mRNA expression of mitochondrial transcription factor 1 (*Tfam*) compared with Tg-NSF mouse hearts, and there was no significant difference between NTg-SF and NSF mouse hearts. In parallel, we observed the upregulation of mitochondrial membrane ATP synthase (*Atp5a*) in Tg-SF mouse hearts compared to NTg-SF mouse hearts, and there was no significant difference between NTg-NSF and SF mouse hearts (Figure 4C).

Interestingly, immunoblotting of lysates obtained from Tg-SF mouse hearts for mitochondrial-related proteins showed significant changes in cytochrome *c* (Cytc) and mitochondrial creatine kinase (CKmito) levels but decreases in the levels of voltage-dependent anion channel (Vdac) with respect to Tg-NSF mouse hearts (Figures 4D and S2).

Mitochondrial-related apoptosis is known to be involved in ventricular remodeling and cardiac dysfunction.^26^^,^^27^ Therefore, we speculated that SF influences the apoptosis process. As expected, upon SF treatment, the mRNA expression levels of BCL2-associated X protein (*Bax*), caspase 3 (*Casp3*), B-cell leukemia/lymphoma 2 (*Bcl2*), and BCL2-associated agonist of cell death (*Bad*) were profoundly increased in Tg-SF mouse hearts compared with Tg-NSF mouse hearts (Figure 4C). Notably, the protein levels of the cleaved caspases Casp9 and Casp3 were significantly higher in Tg-SF mouse hearts (Figure 4E) than in their respective Tg-NSF mouse hearts. None of these parameters showed significant differences when compared between NTg-NSF and NTg-SF mouse hearts. Collectively, these data indicate that SF can cause mitochondrial-related dysfunction and induce apoptosis in mouse hearts.

### SF affects cardiac redox homeostasis

**Figure 5:**
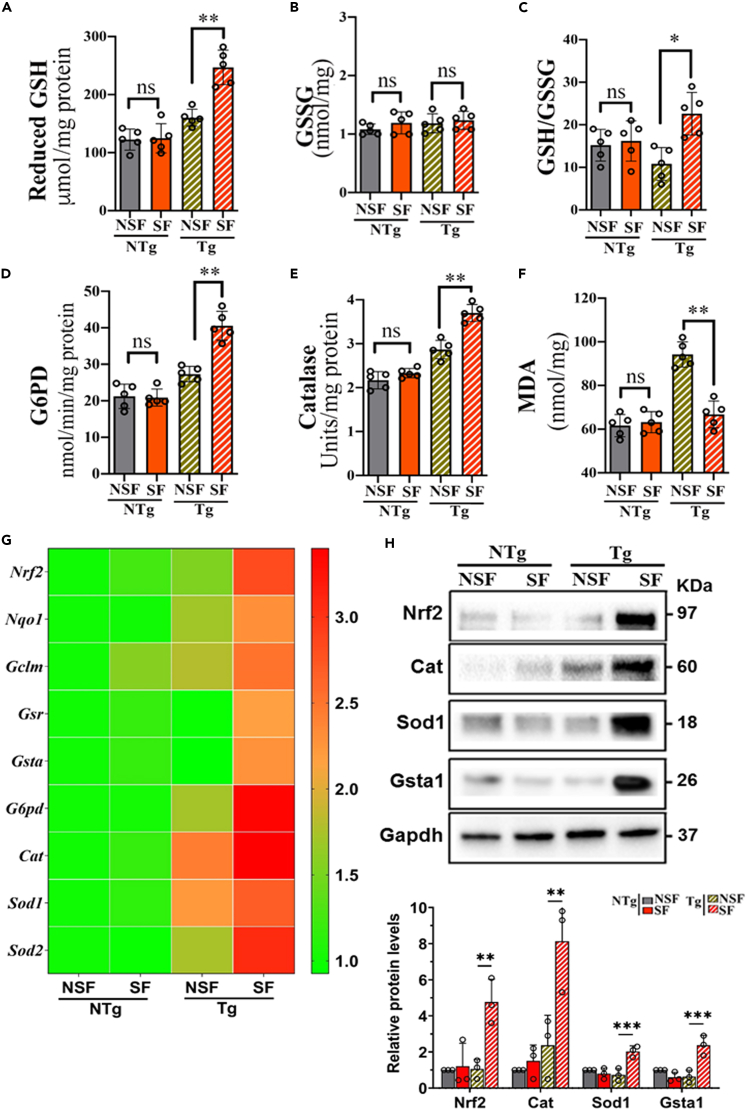
SF alters redox homeostasis (A) Reduced glutathione (GSH) levels, (B) oxidized glutathione (GSSG) levels and (C) GSH/GSSG ratio in mouse heart tissues isolated from NTg-NSF, NTg-SF, Tg-NSF, and Tg-SF, respectively, were measured by enzymatic recycling method. (D) Glucose-6-phosphate dehydrogenase (G6PD) activity, (E) catalase activity and (F) malondialdehyde levels measured in NTg-NSF, NTg-SF, Tg-NSF, and Tg-SF mouse heart tissues. (G) Quantitative RT-PCR analysis of redox regulating genes (*Nrf2*, *Nqo1*, *Gclm*, *Gsr*, *Gsta*, *G6pd*, *Cat*, *Sod1*, and *Sod2*) in NTg-NSF, NTg-SF, Tg-NSF, and Tg-SF mouse heart tissues. mRNA levels were normalized to *Gapdh* and presented as relative expression levels compared to level in NTg-NSF mouse heart tissues. Values are mean ± SEM. with each experiment performed in triplicates (n = 6 in each group). (H) Representative immunoblots with respective proteins from the total lysates of mouse heart tissues isolated from indicated mouse heart tissues. Expression levels were normalized to loading control and presented as relative expression levels compared with the level in NTg-NSF mouse heart tissues. Gapdh levels were used as a loading control. Values are shown as means ± SEM with each experiment performed in triplicate (n = 3 in each group). Significance was evaluated by Student’s *t* test or one-way analysis of variance (ANOVA) with post hoc sidak multiple comparison test, respectively. ∗p < 0.05, ∗∗p < 0.01, and ∗∗∗p < 0.001. See also Figure S2.

Mitochondria also play a crucial role in reactive oxygen species (ROS) generation, which regulates redox balance.^28^ Therefore, we assessed various redox-specific biomarkers in mouse heart lysates from each group. First, we analyzed glutathione (GSH) metabolism and thioredoxin reductase-2 (TrxR2) levels, both play a substantial role in maintaining the myocardial redox balance.^29^^,^^30^ We observed an increase in the concentration of reduced GSH in Tg-SF mouse heart tissue homogenate (Figure 5A), whereas no significant differences were observed in the oxidized form (glutathione disulfide or GSSG) relative to Tg-NSF mice (Figure 5B). In addition, the GSH to GSSG ratio and levels of TrxR2 (Figure S2), reliable indicators of redox status, were significantly greater in Tg-SF mouse hearts than in Tg-NSF mouse hearts. However, SF does not affect GSH levels in NTg mouse hearts. The increase in GSH to GSSG ratio and TrxR2 levels indicate that SF might induce redox stress in Tg-SF mouse hearts (Figures 5C and S2).

Furthermore, the activity of glucose-6-phosphate dehydrogenase (G6PD) (a major cellular source for reducing equivalent NADPH) and catalase (an important hydrogen peroxide scavenger) was higher in Tg-SF mouse hearts than in NTg-SF mouse hearts (Figures 5D and 5E). Notably, in both NTg-SF and NSF mouse hearts, changes in G6PD and catalase activity were not observed (Figures 5D and 5E). In parallel, Tg-SF mouse hearts exhibited lower malondialdehyde (MDA) levels (an indicator of oxidative stress) than Tg-NSF mouse hearts (Figure 5F). However, we did not observe any significant differences in MDA levels between NTg-SF and NTg-NSF mouse heart tissues (Figure 5F).

Next, we tested whether these biochemical changes were reflected at the transcriptional and protein levels in the Tg-SF, Tg-NSF, NTg-SF, and NTg-NSF mouse hearts. We focused on GSH metabolism regulatory pathways, including Nrf2 (nuclear factor erythroid 2-related factor 2)-antioxidant enzymes. We observed that the mRNA expression of *Nrf2* was increased in Tg-SF mouse heart tissues compared with Tg-NSF mouse heart tissues. Concurrently, the expression of the Nrf2 regulator Kelch-like ECH-associated protein 1 (*Keap1*) was decreased upon SF exposure in Tg mouse hearts (Figure 5G). Consequently, Nrf2-associated antioxidant enzyme genes, such as NAD(P)H quinone dehydrogenase 1 (*Nqo1*), glutamate-cysteine ligase, modifier subunit (*Gclm*), glutathione reductase (*Gsr*), glutathione S-transferase, alpha 1 (*GSTα*), glucose-6-phosphate dehydrogenase (*G6pd*), catalase (*Cat*), superoxide dismutase 1, soluble (*Sod1*), and superoxide dismutase 2, mitochondrial (*Sod2*), showed significant upregulation in Tg-SF mouse hearts compared with Tg-NSF mouse hearts (Figure 5G). In contrast, SF exposure had only a modest effect on the expression of antioxidative genes in NTg mouse hearts (Figure 5G). Immunoblotting experiments showed a significant increase in the expression levels of Nrf2 and its associated representative antioxidant enzymes, including Cat, Sod1, and GST*α,* in Tg-SF mouse heart tissues compared with Tg-NSF mouse heart tissues (Figure 5H).

Taken together, these results confirm that SF has a significant influence on cellular redox homeostasis in Tg mouse hearts by increasing the levels of reducing equivalents and augmenting the expression of major antioxidative enzymes.

## Discussion

SF is highly prevalent among various sleep disorders,^10^^,^^31^ and in this study, we explored its consequences on the HCM disease course. Indeed, a significant percentage of HCM patients suffer from sleep apnea.^32^ We chose a genetic HCM model since sarcomere gene variants, such as *MYBPC3* variants, are the leading cause of HCM.^16^^,^^33^ We generated and characterized a humanized transgenic HCM mouse model expressing human-specific *MYBPC3* mutant proteins that displays hypertrophy at five months. After confirming hypertrophy phenotypes (at five months), we subjected the HCM transgenic (Tg) and non-transgenic (NTg) littermates to eight weeks of SF. Interestingly, the cardiac performance was compromised with reduced ejection and FS, suggesting a rapid transition toward dilated or HF phenotypes in the Tg-SF mouse hearts compared to Tg-NSF mouse hearts. In earlier studies, diurnal disturbance was reported to cause adverse cardiac remodeling and had a deleterious effect on survival in myocardial infarction (MI) animal models.^34^ Recent evidence in humans indicates mild to moderate sleep apnea is common among patients with coronary artery diseases with increased LV wall thickness.^35^^,^^36^^,^^37^ In addition, sleep restriction in the MI rat model leads to an enlarged heart size and concomitantly progresses to HF.^38^ In line with these studies, we observed enlarged hearts with increased expression of HF markers, such as *Nppa* and *Nppb*, in Tg-SF mouse hearts compared to Tg-NSF mouse hearts. However, the roles of SF, without sleep restriction, in a genetically driven HCM model have not been studied until now. Taken together, our results reveal a new finding that SF can induce or increase the pace of adverse cardiac remodeling in a genetically induced HCM mouse model of HF.

We employed a model of SF (orbital shakers to rouse mice from sleep every 2 min) that was developed to mimic arousal frequencies experienced during moderate to severe sleep apnea.^39^ It is a version of the Sinton et al. (2009) protocol but without auditory cues.^40^ This method has been shown to consistently fragment sleep without any reduction in total sleep time^39^^,^^41^ indicating that the effect is due to a disruption in the sleep processes as opposed to sleep restriction. Additionally, our results are unlikely to be caused by stress, as this method^39^^,^^41^ and other SF methods^42^ do not increase circulating corticosteroid concentration or elicit behavioral signs of stress. Recently, SF was shown to increase the susceptibility of mice to develop atherosclerosis,^2^ consistent with the deleterious effect on cardiovascular function observed here.

Epidemiologically related human studies suggest that sleep disturbance is strongly associated with adverse cardiovascular disease outcomes by disrupting internal circadian synchronization in shift workers.^43^^,^^44^ Even though clock gene expressions are not significantly altering in acute sleep deprived murine hearts,^45^ the consequences of chronic sleep deprivation are not clear in the heart. In this study, we observed significant modulation of the expression pattern of core clock genes specifically in the chronic SF transgenic mouse hearts. These data suggest that the chronic SF might alter expression of clock-related genes.

Sleep deprivation affects mitochondrial functions in various models.^20^^,^^21^^,^^22^^,^^23^^,^^24^^,^^25^ In clinical setup, OSA patients display severe mitochondrial structure damage and increased mtDNA copy numbers.^46^^,^^47^ In line with this evidence, we observed mitochondrial damage with significantly reduced Vdac1 (an outer mitochondrial membrane protein) levels and an increased CKmito levels in Tg-SF mouse heart tissues. Reduced Vdac1 levels may modulate substrate and ADP flow into mitochondria that may compromise the electron transport chain function and ATP production.^48^ Notably, abnormal CKmito levels were observed in HF patients^49^ and CKmito overexpression mouse models are shown to have initial cardioprotective effects but not in subsequent stages of HF.^50^ Thus increase in CKmito levels in the HF associated with Tg-SF mouse hearts suggest a loss of its cardioprotective effect. Alternatively, the upregulation of CKmito might imply a compensatory mechanism that limits hypertrophic extent and/or progression toward HF.

In addition, mitochondrial-related cell damage occurs through apoptosis by caspase dependent and independent mechanisms.^51^ Available data suggests a caspase dependent apoptotic mechanism in a sleep restricted mice model.^52^ We observed similar evidence in Tg-SF mouse heart tissues by increased levels of caspase proteins such as Cas9 and Cas3.

SF increases ROS levels and upregulates antioxidative response element genes in various models.^53^^,^^54^^,^^55^^,^^56^^,^^57^^,^^58^ For example, SF causes endothelial dysfunction, vascular structural changes, and oxidative stress in mice and humans by activating NADPH oxidase, which is an ROS generator.^9^^,^^55^^,^^56^ Indeed, the function of quality sleep depends on scavenging ROS accumulated during the wake cycle by increasing the efficiency of antioxidant mechanisms.^57^^,^^58^ Furthermore, insufficient sleep alters the expression of oxidative stress genes in human blood cells.^59^

Interestingly, in this study, we observed robust increases in reduced GSH and TrxR2 levels supporting reductive redox stress mechanisms in Tg-SF mouse heart tissues. Additionally, the mRNA expression and protein levels of Nrf2, a master regulator of antioxidant enzymes, were elevated in Tg-SF mouse hearts. Sustained activation of Nrf2 with GSH is well known to increase reducing equivalents, causing reductive stress in various cardiomyopathy models.^60^^,^^61^ Consequently, we observed increases in the expression levels of major Nrf2-regulated genes, such as *Sod1*, *Sod2*, *Cat*, *Gclm*, and *Nqo1*, in SF-treated transgenic mouse hearts. Notably, cardiac-specific overexpression of Sod2 in mice increased the GSH/GSSG ratio and elevated the levels of reducing equivalents in the mitochondria and cytosol.^62^

Sleep deprivation activates the pentose phosphate pathway and enhances G6PD activity in rat heart muscles.^63^ Additionally, G6PD is a major source of intracellular reducing equivalents, such as NADPH. In our study, enhanced G6PD activity further supports the notion that SF triggers reductive stress by increasing reducing equivalents in transgenic mouse hearts. In parallel, SF-Tg mice displayed a significant reduction in MDA levels, indicating lower lipid peroxidation production (an oxidative stress indicator). Therefore, our data support a mechanism in which SF dysregulates redox homeostasis and plays a crucial role in the transition of HCM to HF.

In conclusion, our data suggest that SF induces the transition of HCM toward HF in mice genetically predisposed to HCM. As a mechanistic link, we have demonstrated that SF results in redox stress with mitochondrial damage, sarcomere disarray and apoptosis, subsequently leading to a dilated/HF phenotype. This has an important implication in a clinical setting where the transition of hypertrophy to HF is poorly understood. Our data support SF as a novel risk factor contributing to the progression of HF in HCM patients.

### Limitations of the study

Our study focuses on establishing the pathological role of SF in an HCM model primarily targeting systolic functions. Further studies by measuring diastolic functions will provide additional insights. Also, we provided evidence for caspase-dependent mechanisms in TG-SF mice; however secondary involvement of caspase-independent mechanisms cannot be excluded.

## STAR★Methods

### Key resources table

**Table:** 

REAGENT or RESOURCE	SOURCE	IDENTIFIER
**Antibodies**		

CLOCK (D45B10) Rabbit mAb	Cell Signaling Technology	Cat#5157; RRID:AB_10695411
BMAL1 (D2L7G) Rabbit mAb	Cell Signaling Technology	Cat#14020; RRID:AB_2728705
Cytochrome *c* (136F3) Rabbit mAb	Cell Signaling Technology	Cat#4280; RRID:AB_10695410
VDAC (D73D12) Rabbit mAb	Cell Signaling Technology	Cat#4661; RRID:AB_10557420
Cleaved Caspase-9 (Asp330) (E5Z7N) Rabbit mAb	Cell Signaling Technology	Cat#52873; RRID:AB_2799423
Cleaved Caspase-3 (Asp175) (5A1E) Rabbit mAb	Cell Signaling Technology	Cat#9664; RRID:AB_2070042
Anti-NRF2 (D1Z9C) XP Rabbit monoclonal Antibody	Cell Signaling Technology	Cat#12721; RRID:AB_2715528
Catalase (D4P7B)XP® Rabbit mAb	Cell Signaling Technology	Cat#12980; RRID:AB_2798079
GST-Tag (26H1) Mouse mAb	Cell Signaling Technology	Cat#2624; RRID:AB_2189875
GAPDH (D16H11) XP Rabbit mAb	Cell Signaling Technology	Cat#5174; RRID:AB_10622025
Anti-PER1 antibody - N-terminal	Abcam	Cat#ab136451; RRID:AB_3075441
Anti-mCherry antibody	Abcam	Cat# ab167453; RRID:AB_2571870
Anti-Creatine kinase MT antibody	Abcam	Cat#ab198257, RRID:AB_3075442
TrxR2 Recombinant Rabbit Monoclonal Antibody (ARC1339)	ThermoFisher Scientific	Cat#MA5-35730, RRID:AB_2849630
ANTI-MYBPC3 (N-TERM) antibody	Sigma-Aldrich	Cat#SAB1303629-400UL; RRID:AB_3075444

**Chemicals, peptides, and recombinant proteins**		

Hydrogen peroxide solution	Sigma	Cat#88597
Phosphate Buffer Solution	Sigma	Cat#P3619
2-Thiobarbituric acid	Sigma	Cat#T5500
Glutaraldehyde solution	Sigma	Cat#G5882

**Critical commercial assays**		

Glutathione Assay Kit	Cayman chemical	Cat#703002
Bio-Rad Protein Assay Kit II	Biorad	Cat#5000002
Glucose-6-Phosphate Dehydrogenase Assay Kit (Colorimetric)	Abcam	Cat#ab102529
Aurum™ Total RNA Mini Kit	Biorad	Cat#7326820
iScript™ Reverse Transcription Supermix for RT-qPCR	Biorad	Cat#1708841
Pierce™ BCA Protein Assay Kits	ThermoFisher Scientific	Cat# 23225

**Experimental models: Organisms/strains**		

Humanized MYBPC3 mice on C57BL/6 background	This paper	N/A

**Oligonucleotides**		

See Table S1 for primers sequences	This paper	N/A

**Software and algorithms**		

GraphPad Software	San Diego, CA	https://www.graphpad.com/
ImageJ	National Institutes of Health (NIH)	https://imagej.net/ij/

### Resource availability

#### Lead contact

Further information and requests for resources and reagents should be directed to and will be fulfilled by the lead contact, Perundurai S Dhandapany (dhan@instem.res.in).

#### Materials availability

Materials generated in this study are available from the lead contact upon request.

#### Data and code availability

• All data reported in this paper will be shared by the lead contact upon request.
• This paper does not report original code.
• Any additional information required to reanalyze the data reported in this paper is available from the lead contact upon request.

### Experimental model and study participant details

#### Generation and characterization of transgenic humanized mouse models

Humanized transgenic mice lines were generated using a recombinant plasmid construct containing human (patient) MYBPC3 cDNA with modified C10 domain downstream of a mouse alpha-myosin heavy chain (α-MHC) promoter (Figure 1A). Restriction digestion and subsequent sequencing analysis confirmed orientation and integrity of the inserts. Two transgenic founder lines were established based on their hMYBPC3 expression levels. Transgenic founder mice were further back-crossed with C57BL/6 background for at least 6 generations. Transgenic mice were genotyped and confirmed by PCR using the specific primers: GAAGTGGTGGTGTAGGAAAGTCAG and CTTGTTGCTGGCGCTGATGTC. Protein expression analysis of hMYBPC3 (mCherry) transgene was done in comparison with total Mybpc3 in transgenic mice hearts.

Tg mice with relatively high expression (Tg-high) were not used due to severe mortality rate. The minimal or low Tg expression lines (designated as Tg) were further expanded and used for subsequent experiments. The age and sex matched littermates without transgene were considered as non-transgenic (NTg) groups. Mice were housed under a controlled temperature and humidity, a 12 h light/dark cycle, and fed with a standard rodent diet and water *ad libitum*. For sleep fragmentation experiments, we used five months old male and female mice (n = 3–4 per cage). Animals were handled as approved by the respective Institutional Animal Care and Use Committees in accordance with the “Principles of Laboratory Animal Care by the National Society for Medical Research and the Guide for the Care and Use of Laboratory Animals” (NIH Publication No. 86-23, revised 1996).

### Method details

#### Sleep fragmentation experiments

To study the effects of sleep fragmentation, mice were group-housed (n = 3–4 per cage) in Thoren #1 acrylic cages (19.6 cm × 30.9 cm × 13.3 cm) with pelleted cellulose bedding (BioFresh Performance Bedding, 1⁄4″ pelleted cellulose, Absorption Corp, Ferndale, WA). Both males and females were studied. Mice were housed in light-tight cabinets (Phenome Technologies) with a 12L:12D light schedule provided by green LEDs (525nm, 125 lux). General activity of the cages was recorded by passive infrared sensors (Telos Discovery Systems).^64^^,^^65^ Total movement of the group-housed mice was collected as counts per 10 min bin and visualized in actograms using ClockLab (Actimetrics, Wilmette, IL).

Sleep was fragmented in the mice chronically for 8 weeks by placing the cages on orbital shakers (Lab-Line 3520, Lab-Line Instruments, Melrose Park, IL) that were connected to timers that set them to oscillate periodically (10 s at 110 rpm followed by 99 s of rest). This non-invasive technique has been used in mice and voles.^39^^,^^40^^,^^41^ Though mice react in the first few days with decreases in sleep,^40^^,^^39^ this dissipates and in chronic conditions, mice exhibit normal sleep duration but with increased arousal index and a small reduction in total REM sleep.^39^ Importantly, there is no increased stress as measured by circulating corticosterone.^39^ During the 8-week experiment, mice were provided with food (Lab Diet 5L0D) *ad libitum*; hydration was provided by HydroGel (ClearH2O, Westbrook, ME), because water bottles will leak during the shaking periods. Body weight was recorded weekly for the duration of the sleep fragmentation study.

#### Mouse echocardiography and tissue collection

Experimental mice were anesthetized with 1% isoflurane in oxygen in a sealed plastic chamber. Then the immobile mice were transferred onto a board and nose cone connected with 1% isoflurane vaporizer during the entire procedure. The scan head was placed on the chest of the mouse, and stable image signals (both B mode and M mode) were acquired and data analyzed with Vevo 770 (Visual Sonics). Systolic and diastolic left ventricle peripheral wall thickness, chamber diameter, and interventricular wall thickness were measured with M mode image. All the analyses were performed offline using a workstation equipped with the Vevo lab software – V3.1.0 (FUJIFILM VisualSonics, Japan). All measurements were averaged over six cardiac cycles. Left ventricular (LV) M-mode at the mid-papillary level measurements of the size of the LV walls and cavities were obtained by 2D guidance from the short-axis view of the LV. LV internal diameter, LV posterior wall thickness, and LV anterior wall thickness were measured at end diastole. LV mass was derived by cubic method. In addition, LV systolic diameter was measured and LV fractional shortening and LV ejection fraction were calculated.^66^ All mice were euthanized and perfused between zeitgeber times 3 and 7 (ZT3-7; by convention ZT0 and ZT12 are defined by lights-on and lights-off). The samples were immediately used or frozen at −80°C for further analyses based on the assays performed.

#### Biochemical assays

Measurement of glutathione level: Myocardial levels of total and oxidized GSH were assessed using Glutathione assay kit (Cayman, USA) following manufacturer instructions. In brief, MES buffer (0.4 M 2-(N-morpholino) ethanesulphonic acid, 0.1 M phosphate and 2mM EDTA, pH 6.0) was used to prepare the cardiac homogenates and centrifuged at 5000 rpm for 5 min at 4°C. Supernatant was used for protein determination and equal amount of 10% *meta*-phosphoric acid (MPA) was added to the remaining samples to precipitate the proteins. To adjust the pH for total glutathione quantification, the MPA extracts were treated with triethanolamine (TEAM). An aliquot of TEAM treated extract (100 μL) was mixed with 150 μL of reaction mixture cocktail (containing MES buffer, nicotinamide adenine dinucleotide phosphate (NADPH), Glutathione Reductase enzyme and 5,5′-dithio-bis (2-nitrobenzoic acid) (DTNB)) and immediately measured the kinetics of enzymatic-recycling assay at 412 nm using a plate reader. For GSSG measurement, another aliquot of TEAM treated extract (100 μL) was treated with 2-vinyl pyridine and incubated for 1.0 h at room temperature. After incubation, 150 μL of reaction mixture cocktail was added and immediately measured the kinetics of enzymatic-recycling assay at 412 nm. Similarly, GSH and GSSG standards were treated and measured to obtain a standard graph to extrapolate the values. The concentration of reduced glutathione (GSH) was estimated by subtracting the measured oxidized (GSSG) glutathione levels from the measured total glutathione (GSH plus GSSG). GSH/GSSG ratio was then determined.

Catalase activity: Catalase activity was measured in the cardiac tissue lysates by the spectrophotometric method. Briefly, tissue homogenate was added to a cuvette containing 50 mM phosphate buffer, pH 7.4, and the reaction was started by addition of 1.0 mL of freshly prepared 30 mM H2O2. Measurements were calculated at 240nm and performed in triplicate. Protein concentrations were estimated by the Bradford method. Catalase activity was calculated as units per milligram of protein.

Glucose-6-phosphate dehydrogenase (G6PD) activity: G6PD activity in the cardiac tissue lysates were determined by measuring reduced nicotinamide adenine dinucleotide (NADH) at 450 nm using a glucose-6-phosphate dehydrogenase assay kit (ab102529, Abcam) according to the manufacturer’s instructions. The protein concentration was measured for each sample, and enzyme activity was calculated using a reduced NADH standard curve and is expressed as nmol/min/mg protein.

Lipid peroxidation: Malondialdehyde (MDA) levels were evaluated in the cardiac tissue lysates using thiobarbituric acid (TBA) to produce MDA-TBA complex at 90°C − 100°C, a color change was measured at 532 nm with a spectrophotometer.

#### Quantitative real-time PCR

Cardiac tissues were homogenized and RNA was extracted using Bio-RAD kit. Then, the cDNA was synthesized using 1.25 μg RNA and iScript Reverse Transcription Supermix for quantitative real-time PCR (qPCR; Bio-Rad). cDNA was amplified using the following PCR conditions: 95°C for 2 min, followed by 40 cycles of 95°C for 15 s and 60°C for 1 min and primers used were given in Table S1. Data were analyzed using the 2−ΔΔCT method. Fold changes in gene expression were determined using the relative comparison method with normalization to glyceraldehyde-3-phosphate dehydrogenase (Gapdh).

#### Immunoblot analysis

Total proteins were extracted from the cardiac tissues were homogenized in urea buffer (4M urea, 1 M thiourea, 50 mM Tris-HCl, pH 7.5, 0.4% (w/v) CHAPS, 20mM spermine, and 20 mM DTT) containing protease (Roche 4693159001) and phosphatase inhibitors (Sigma P5726 and P0044).^17^ Protein concentrations of samples were then measured by a Pierce BCA Protein Assay Kit (ThermoFisher Scientific). Fifty micrograms of protein samples were subjected to SDS-PAGE (Invitrogen), and then were transferred to a polyvinylidene difluoride membrane (Millipore). After blocking with 5% non-fat milk in Tris-buffered saline, membranes were hybridized overnight at 4°C with primary antibodies. Following primary antibodies were used: Clock (Cat No. 5157, Cell Signaling Technology), Bmal 1 (Cat No. 14020, Cell Signaling Technology), Cytochrome *c* (Cat No. 4280, Cell Signaling Technology), Vdac (Cat No. 4661, Cell Signaling Technology), Cleaved caspase 9 (Cat No. 52873, Cell Signaling Technology), Cleaved caspase 3 (Cat No.9664, Cell Signaling Technology), Nrf2 (Cat No. 12721, Cell Signaling Technology), Catalase (Cat No. 12980, Cell Signaling Technology), Gst (Cat No. 2624, Cell Signaling Technology), Gapdh (Cat No. 5174, Cell Signaling Technology), Per1 (Cat No. ab136451, abcam), mCherry (Cat No. ab167453, abcam), TrxR2 (Cat No. MA5-35730, ThermoFisher Scientific), CKmito (Cat No. ab198257, abcam) and Mybpc3 (Cat No. SAB1303629, Sigma). The membranes were then incubated with peroxidase-conjugated secondary antibodies (Jackson ImmunoResearch Laboratories, at 1:10 000 dilution), and the protein signals were detected with the ChemiDoc TM XRS+ (Bio-Rad) system (Bio-Rad, Hercules, CA, USA). Protein expression levels were normalized to corresponding Gapdh levels.

#### Electron microscopy studies

The organelle changes were analyzed using transmission electron microscopy (TEM). Mice were anesthetized with isoflurane and hearts were fixed by perfusion with 3.5% glutaraldehyde in cardioplegic buffer (100 mM KCl, 5% dextrose in PBS) for 2 min, followed by 3.5% glutaraldehyde in 100 mM cacodylate buffer (pH 7.3) for 2 min. The fixatives were gravity fed (600 mm) into the hearts through the apex and right ventricle. Left ventricular cardiac tissue samples were dissected and then fixed with 2.5% glutaraldehyde for 18h and 4% formaldehyde in 0.1 M HEPES buffer. Following this, the tissues were further fixed with 1% osmium tetroxide in 0.1 M sodium cacodylate buffer for 1 h at 4°C, dehydrated in graded alcohol and then embedded in Epon (Polysciences, Inc. Warrington, PA, USA). Ultrathin sections were cut with a diamond knife on a Leica UC7 Ultramicrotome. Thin sections were counter stained with uranium and lead salt and examined on a transmission electron microscope. The distance between two thick myosin filaments (sarcomeric length) were measured for tissue sections from each mice group. Sarcomeric length was measured using ImageJ and converted into micrometre. Abnormal mitochondria were defined by hypertrophied and rounded structures and were counted and represented in percentage.

### Quantification and statistical analysis

Differences between genotypes were assessed by two-tailed Student’s *t* test and one-way analysis of variance with appropriate post hoc adjustment. A value of p < 0.05 and below was considered to be statistically significant.
