# Supplementary material for: Sleep fragmentation induces heart failure in a hypertrophic cardiomyopathy mouse model by altering redox metabolism
Source: iScience. 2024 Feb 1;27(3):109075. doi: 10.1016/j.isci.2024.109075 (PMC10867644; doi:10.1016/j.isci.2024.109075)
Supplement: Document S1. Figures S1 and S2 and Table S1 [file mmc1.pdf]

iScience, Volume *27*

Supplemental information

Sleep fragmentation induces heart failure in a hypertrophic cardiomyopathy mouse model by altering redox metabolism

Karthikeyan Bose, Radhika Agrawal, Thiagarajan Sairam, Jessenya Mil, Matthew P. Butler, and Perundurai S. Dhandapany

Table S1, related to STAR Methods. Primers used for qRT-PCR

Gene Name	Forward Primer	Reverse Primer
Nppa	GCTTCCAGGCCATATTGGAG	GGGGGCATGACCTCATCTT
Nppb	GAGGTCACTCCTATCCTCTGG	GCCATTTCCTCCGACTTTTCTC
Pln	GGACCAAAGGAACTTGCCAGCT	CAACAGGCAGCCAAATGTGAGC
Clock	GGCTGAAAGACGGCGAGAACTT	GTGCTTCCTTGAGACTCACTGTG
Arntl	ACCTCGCAGAATGTCACAGGCA	CTGAACCATCGACTTCGTAGCG
Cry 1	GGTTGCCTGTTTCCTGACTCGT	GACAGCCACATCCAACTTCCAG
Cry 2	GGACAAGCACTTGGAACGGAAG	ACAAGTCCCACAGGCGGTAGTA
Per 1	GAAACCTCTGGCTGTTCCTACC	AGGCTGAAGAGGCAGTGTAGGA
Per 2	CTGCTTGTTCCAGGCTGTGGAT	CTTCTTGTGGATGGCGAGCATC
Per 3	CACAGACATCGAAGGAGGTGCT	CTTACACGCCACGGCAACACTT
Tfam	GAGGCAAAGGATGATTCGGCTC	CGAATCCTATCATCTTTAGCAAGC
Atp5a1	TGGTGAAGAGACTGACGGATGC	TCAAAGCGTGCTTGCCGTTGTC
Bax	AGGATGCGTCCACCAAGAAGCT	TCCGTGTCCACGTCAGCAATCA
Casp3	GGAGTCTGACTGGAAAGCCGAA	CTTCTGGCAAGCCATCTCCTCA
Bcl2	CCTGTGGATGACTGAGTACCTG	AGCCAGGAGAAATCAAACAGAGG
Bad	GGGAGCAACATTCATCAGCAGG	CGTCCTCGAAAAGGGCTAAGCT
Nrf2	CTGAACTCCTGGACGGGACTA	CGGTGGGTCTCCGTAAATGG
Nqo1	AGGATGGGAGGTACTCGAATC	TGCTAGAGATGACTCGGAAGG
Gclm	CTTCGCCTCCGATTGAAGATG	AAAGGCAGTCAAATCTGGTGG
Gsr	CACGGCTATGCAACATTCGC	GTGTGGAGCGGTAAACTTTTTC
Gst	TACTTTGATGGCAGGGGAAG	TCATCCCGTCGATCTCTACC
G6pd	CCGGAAACTGGCTGTGCGCT	CCAGGTCACCCGATGGCACCC
Cat	CGGCACATGAATGGCTATGGATC	AAGCCTTCCTGCCTCTCCAACA
Sod1	AACCAGTTGTGTTGTCAGGAC	CCACCATGTTTCTTAGAGTGAGG
Sod2	TGGACAAACCTGAGCCCTAAG	CCCAAAGTCACGCTTGATAGC
Gapdh	TGACCTCAACTACATGGTCTACA	CTTCCCATTCTCGGCCTTG

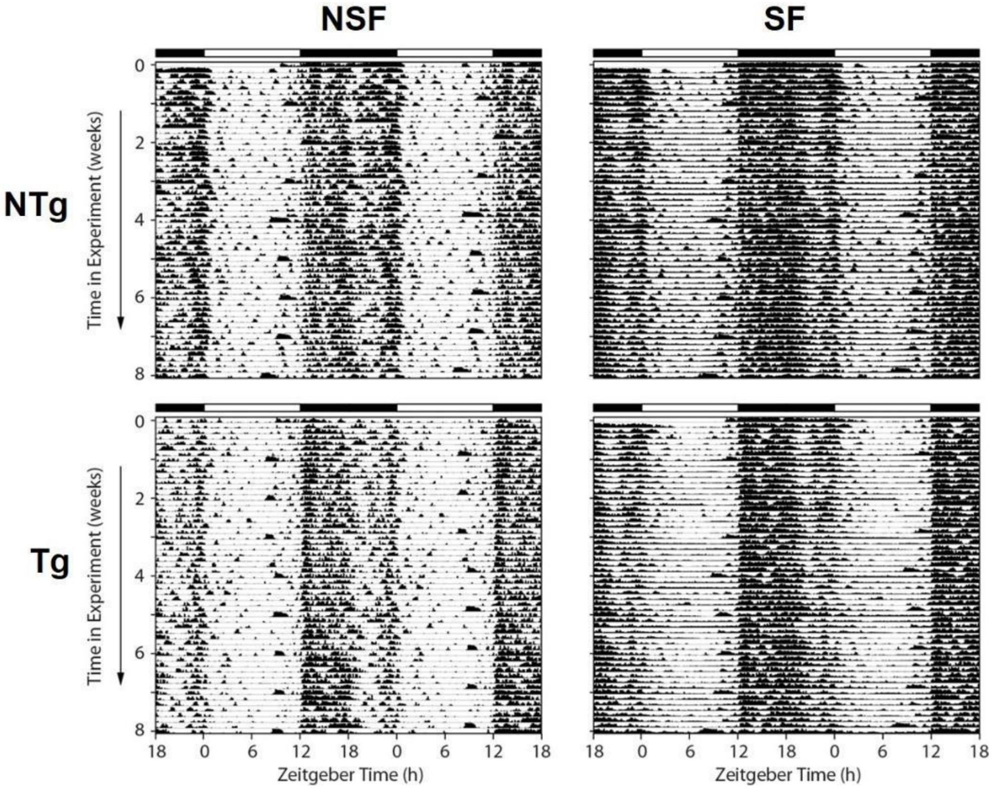

Figure S1, related to Figure 2. Representative actograms of NTg and Tg with and without sleep fragmentation (Shaking). Double-plotted actograms of locomotor activity, collected in 10 min bins by passive infrared detectors above the cages. Days are plotted consecutively from top to bottom, and hatch marks along each line indicate the amount of activity (normalized to the maximum activity within each day, ClockLab). All mice were entrained similarly to the light-dark cycle (shown above each actogram; lights on ZT0 to ZT12). Shaking every two minutes led to an increase in baseline activity, observable throughout the light phase during sleep fragmentation. Larger bouts of activity during the day once per week correspond to cage changes.

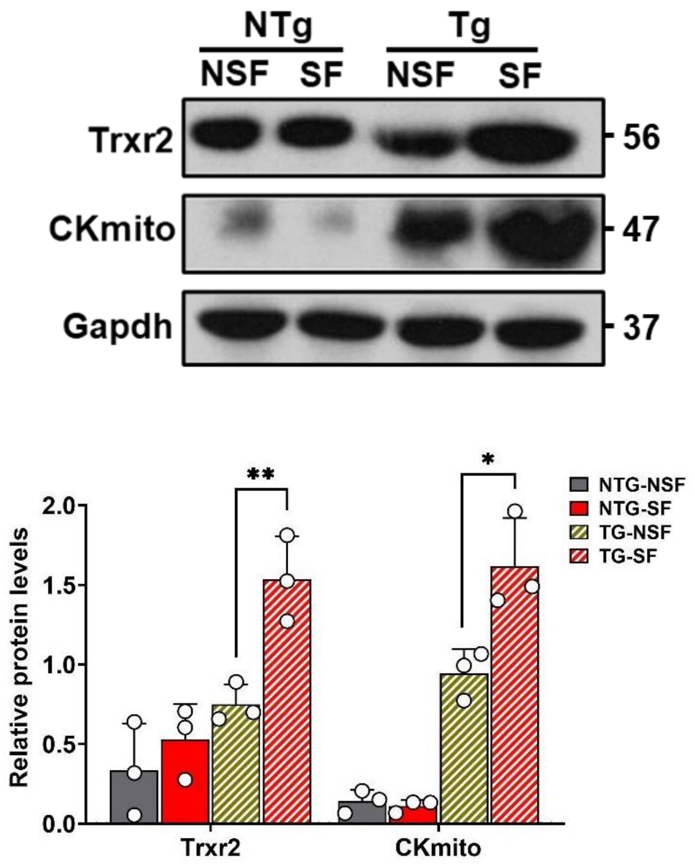

Figure S2, related to Figures 4 and 5. SF alters the expression of thioredoxin reductase and mitochondrial creatine kinase

Representative immunoblots with respective proteins from the total lysates of mouse heart tissues isolated from NTg-NSF, NTg-SF, Tg-NSF and Tg-SF mouse heart tissues. Expression levels were normalized to loading control and presented as relative expression levels compared with the level in NTg-NSF mouse heart. Gapdh levels were used as a loading control. Values are shown as means±SEM with each experiment performed in triplicate (n=3 in each group). Significance was evaluated by two-way analysis of variance (ANOVA) with post hoc sidak multiple comparison test, respectively. *p<0.05 and **p<0.01.
